# Optimal Sparse Energy Sampling for X-ray Spectro-Microscopy: Reducing the X-ray Dose and Experiment Time Using Model Order Reduction

**DOI:** 10.1021/cbmi.3c00116

**Published:** 2024-03-19

**Authors:** Paul D. Quinn, Malena Sabaté Landman, Tom Davis, Melina Freitag, Silvia Gazzola, Sergey Dolgov

**Affiliations:** † Scientific Computing, Science and Technology Facilities Council, Rutherford Appleton Laboratory, Harwell Campus, Didcot OX11 0QX, United Kingdom; ‡ Department of Mathematics, Emory University, Atlanta, Georgia 30322, United States; § Department of Mathematical Sciences, University of Bath, Bath BA2 7AY, United Kingdom; ∥ Institute of Mathematics, University of Potsdam, Karl-Liebknecht-Str. 24-25, 14476 Potsdam, Germany

**Keywords:** X-ray spectro-microscopy, sparse, low-dose, XANES, ptychography, reduced-order model

## Abstract

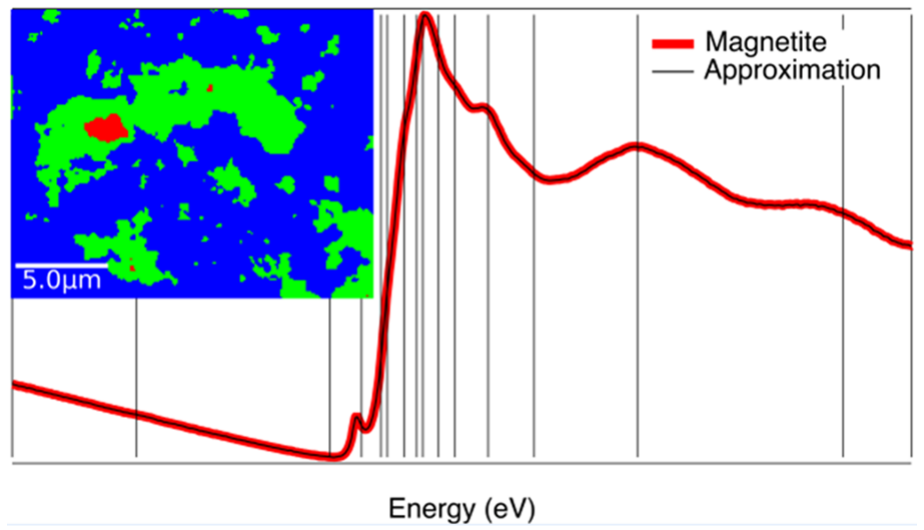

The application of X-ray spectro-microscopy to image changes in the chemical state in application areas such as catalysis, environmental science, or biological samples can be limited by factors such as the speed of measurement, the presence of dilute concentrations, radiation damage, and thermal drift during the measurement. We have adapted a reduced-order model approach, known as the discrete empirical interpolation method, which identifies how to optimally subsample the spectroscopic information, accounting for background variations in the signal, to provide an accurate approximation of an equivalent full spectroscopic measurement from the sampled material. This approach uses readily available prior information to guide and significantly reduce the sampling requirements impacting both the total X-ray dose and the acquisition time. The reduced-order model approach can be adapted more broadly to any spectral or spectro-microscopy measurement where a low-rank approximation can be made from prior information on the possible states of a system, and examples of the approach are presented.

## Introduction

X-ray absorption spectroscopy (XAS) is a powerful technique that can be used to obtain information about the chemical state and electronic and structural properties of materials. This is achieved by using an X-ray beam of variable energy to probe the binding energy of electrons in specific electronic shells of the element (X-ray absorption near edge structure; XANES) or the elastic scattering processes between the photoelectrons generated by the incident beam and other atoms in the vicinity of the absorbing atom (extended X-ray absorption fine structure, EXAFS).

The use of focused beams or coherent imaging techniques such as ptychography, using both hard and soft X-rays, has expanded the application of this technique to also examining spatial variations in the chemical state with resolutions down to a few nanometers to tens of nanometers.^1−3^ Depending on the technique used, the absorption and/or the phase, which are connected via the Kramers–Kronig relation,^4^ are imaged at a series of energies across the absorption edge that, when combined, provide an array of spatially resolved spectra. These spatial mappings of the chemical state have, to date, had limited application to *in situ* studies due to the acquisition times required. The dose required to achieve a reasonable signal-to-noise ratio for XAS and the associated detection sensitivity requirements of the technique have also hampered spectroscopic studies in dilute systems, such as the studies of metal nanoparticles inside cells.

To get around some of these limitations, researchers have looked to reduce the dose and/or time of an individual scan or a stack of scans by employing new hardware approaches to the experiment and new methods to reduce the number of spatial samples or energy samples. To reduce the measurement times for individual maps, fast detectors with integrated approaches to scanning and event processing^5^ can be used to efficiently scan a sample, although the signal-to-noise requirements for XANES from a multi-energy stack of images will still define the limit of the measurement time. Novel artificial intelligence (AI)-driven scanning using route optimization^6^ has been shown to reduce overall time by sparsely sampling and inpainting to reduce measurement time down to 10% in some cases; while this is a promising direction, the application to XANES imaging has not yet been demonstrated. Random spatial subsampling at each energy can exploit the low number of unique chemical states in a real sample to complete missing information and provide a 5- to 6-fold reduction in dose and time.^7^ These approaches are generalized and do not require or exploit any prior knowledge of the system. Knowledge of the possible chemical states should allow for optimized experiment design, significantly reducing the sampling conditions and the dose, and in the best case, the number of energy samples will approach the number of unique chemical states.^8^ The most common approach to reducing the number of energy samples is to use linear combination fitting of selected energy points.^9^ The linear combination fitting (LC) method exploits the additive nature of absorption, with the total absorption of a signal measured being the sum of the absorption of the individual quantities of chemical species present. Assuming a known set of possible components, a few characteristic energies can be manually selected based on some spectral difference or contrast point, such as the shift in peak positions, and the resulting measurements at these points are then manipulated using linear combination fitting to determine the chemical state mixing.^9−11^ However, in practice, the wider use of this approach can be impacted by several factors. The selection of the points to sample in the LC selection is based on a few qualitative or subjective decisions based on perceived contrast points and may not provide optimal contrast. Quadratic or cubic variations in the background of the pre- and post-absorption edge regions are not well incorporated in this approach and will impact the accuracy of any resulting LC fit to the reduced number of sample measurements. In phase-based spectro-microscopy, normalizing the data for fitting is not straightforward compared to conventional XAS, and quadratic and cubic backgrounds will play a role in the fitting. The LC approach largely relies on reference standards and does not readily incorporate spectro-microscopy information. New approaches are needed that can optimally reduce sampling across spectro-microscopy techniques, maximize the use of prior information, and incorporate experimental variations to improve results.

Independently, subsampling approaches have been developed to reduce the computational effort in large complex nonlinear models, such as those used in fluid simulations. One such approach, the discrete empirical interpolation method (DEIM), is a deterministic technique first introduced by Chaturantabut and Sorensen,^12^ which reduces complex nonlinear models by approximating the system from snapshots or measurements of its different states and projecting onto a lower-dimensional subspace using proper orthogonal decomposition (POD)^13^ or a similar low-rank determination technique. The nonlinear model is then only evaluated at optimally selected sampling points, and all other model values are approximated via interpolation using the low-rank description of the system.

To design an experimental analogue of this method in the setting of sampling for X-ray absorption spectroscopy, an overall description of the system first needs to be available, which needs to capture the possible states of the system; i.e., a reduced or low-rank description of the system under investigation needs to be developed. For spectro-microscopy, this can be based on fully sampled prior measurements or representative bulk XAS standards that are either specific to the experiment or from a database of standards, along with simulated background variations to ensure the experimental measurement is properly captured. This set of known states or spectra from spectro-microscopy data or from large sets of bulk XAS measurements is then approximated by a low-rank matrix to achieve this reduced description.

Adapting this technique to subsample XAS or spectro-microscopy allows us to (i) optimally select a small set of sampling points based on maximum information content, (ii) address sampling of noisy data and varying backgrounds, (iii) broadly apply this approach across techniques such as spectro-ptychography or traditional XAS, and (iv) use a broad selection of prior information and adapt the sampling information.

## Method

The mathematical description of the method is straightforward. In brief, the method uses existing dimensionality reduction techniques such as PCA (principal component analysis) and SVD (singular value decomposition) to provide a reduced-order subspace before then applying the DEIM method to determine a subset of spectroscopic energies that capture the largest statistical variation between XAS spectra in those reduced-order subspaces.

In detail, let *A*′ ∈ *R*^*m* × *n*′^ represent an overall description of the *n*′ spectra at *m* energy points we could possibly measure or find in the material under inspection, and let *A* ∈ *R*^*m* × *n*^ represent the experiment we wish to perform on a material, which would result in a full description obtained by performing a full scan from *n* spatial positions. The rows and columns of *A*′ and *A* encode energy and spatial information, respectively. We assume that *n*′ < *n* (or even *n*′ ≪ *n*), i.e., the number of distinct chemical states we expect to find is much less than the number of measured spectra. However, for simplicity, the profile of energy points *m* is assumed to be the same. To start, a reduced description of the system, subspace matrix *U*_*k*_ ∈ *R*^*m* × *k*^ with *k* ≤ min(*m*, *n*′), needs to be extracted using only prior information and/or previous scans *A*′. For this step, we can use the SVD of *A*′, which is similar to POD in model order reduction and PCA in signal processing. To this end, we compute
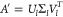
1where *U*_*l*_ ∈ *R*^*m* × *l*^ and *V*_*l*_ ∈ *R*^*n*′ × *l*^ are matrices of left and right singular vectors, respectively, ∑_*l*_ ∈ *R*^*l* × *l*^ is a diagonal matrix of singular values, and *l* = min(*m*, *n*′). Now, we choose a rank value *k* ≤ *l* that will be our modeling parameter (for example, *k* can be set equal to the assumed number of different material chemical states in the sample) and truncate the SVD to *U*_*k*_ ∈ *R*^*m* × *k*^ by selecting only *k* leading columns from *U*_*l*_. Each column represents the source of variation in the experimental data with the first column representing the greatest common variation across the data set. In PCA, *k* reflects the explained variance in the model, and usually, k ≪ *m*, justifying a good low-rank approximation of *A*′.

We then seek an approximation *U*_*k*_*C* ≈ *A*, where *U*_*k*_ ∈ *R*^*m* × *k*^ is a given subspace matrix (obtained, for instance, as described above) and *C* ∈ *R*^*k* × *n*^ is a matrix of coefficients to be computed. Instead of picking *C* to minimize the spectral norm of (*A* – *U*_*k*_*C*), DEIM constructs *C* so that *U*_*k*_*C* interpolates columns of *A* at certain strategically chosen indices. Let *e*_*j*_ denote the *j*th column of the *m* × *m* identity matrix. We impose the interpolation condition at the *k* indices *p*_1_,...,*p*_*k*_ (these are distinct integers between 1 and *m*) to be determined. The submatrix of the identity matrix, *P*: = [*e*_*p*_1__,...,*e*_*p*_*k*__] ∈ *R*^*m* × *k*^, can then be used to extract entries *p*_1_,...,*p*_*k*_ of the columns of *A*, e.g., *e*_*p*_1__^T^*A* = *A*_*p*_1__ and so on. To construct *C* so that *U*_*k*_*C* interpolates *A* at the entries *p*_1_,...,*p*_*k*_, we therefore require *P*^*T*^*A* = *P*^*T*^*U*_*k*_*C*. We then find
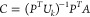
2which provides the value of *C* to approximate *A*. However, the accuracy of the approximation *U*_*k*_*C* depends crucially on the indices *p*_1_,...,*p*_*k*_.

**Figure 1:**
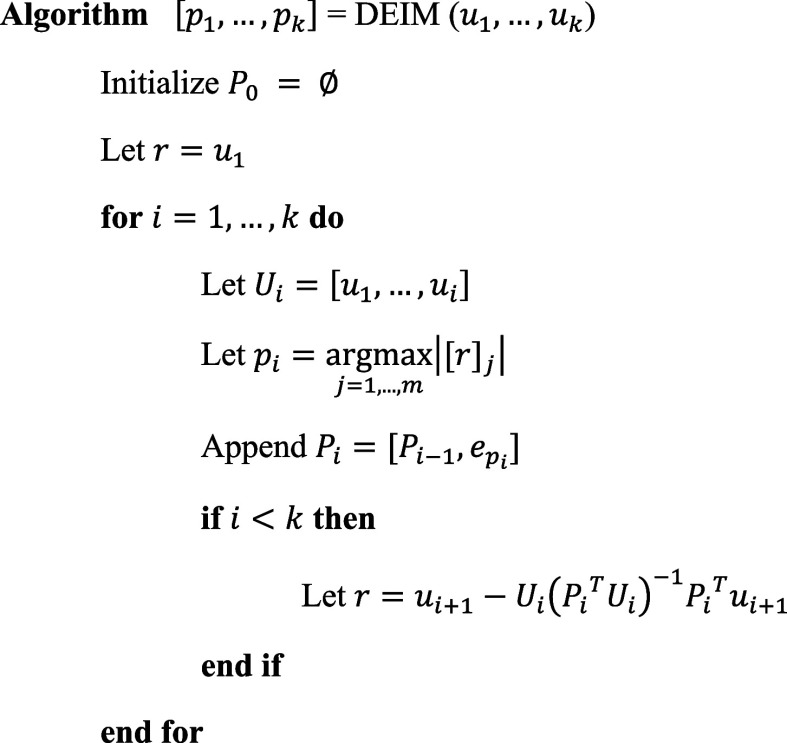
Outline of the DEIM iteration to select the optimal sampling points from a set of orthogonal projections extracted from known standards or prior experimental data.

To select good indices *p*_1_,...,*p*_*k*_, the DEIM algorithm proceeds as follows. Since the columns *u*_1_,...,*u*_*k*_ ∈ *R*^*m*^ of *U*_*k*_ are often ordered by decreasing importance (for example, when computed via an SVD as described above), *u*_1_ is the most important basis vector. DEIM picks its first index *p*_1_ to be the largest magnitude entry in *u*_1_. Defining *P*_1_: = [*e*_*p*_1__] ∈ *R*^*m*^, we then have that Π_1_ = *U*_1_(*P*_1_^*T*^*U*_1_)^−1^*P*_1_^T^ is the interpolatory projector associated with the first index. Defining the residual as *r* = *u*_2_ – Π_1_*u*_2_, where Π_1_*u*_2_ is an approximation to *u*_2_ in the subspace spanned by *u*_1_, DEIM then picks *p*_2_ as the index *j* of the largest entry in magnitude |[*r*]_*j*_|, *j* = 1*,...,m* of the residual *r* and defines *P*_2_: = [*P*_1_, *e*_*p*_2__] ∈ *R*^*m* × 2^. This index selection process is iterated until *k* indices are found, as shown in Figure 1.

The use of PCA on the data source also removes the potential impact of highly correlated variables or multicollinearity, which may impact the conventional LC approach, as PCA reduces the inputs to a set of uncorrelated (orthogonal) principal components.

When implementing this method with different data sources, the primary question is what value of *k*, the number of sampling points, should we select? For a database of independent standards, the value of *k* should be at least equal to the number of standards used to differentiate them accurately. For a set of previously measured spectro-microscopy data, the value of *k* can be selected from the elbow point of the explained variance,^14^ i.e., where additional *k* points do not significantly improve the fit to the existing data.

## Results and Discussion

**Figure 2:**
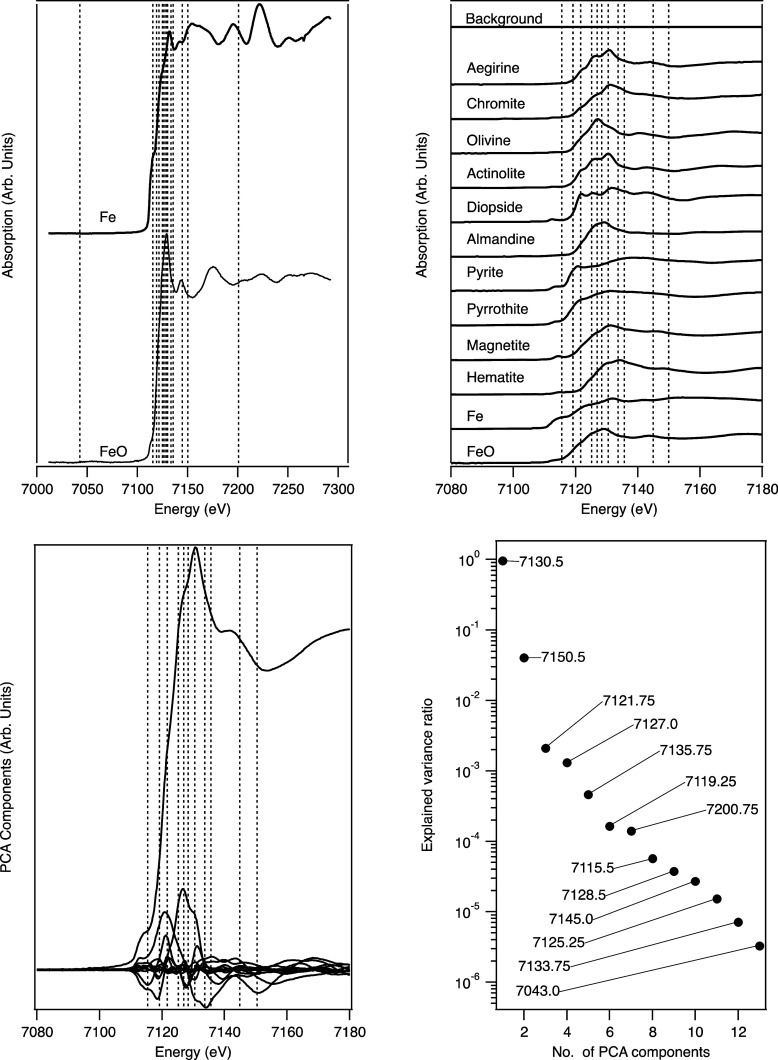
Left to right, top to bottom: (a) Full XANES standard spectra from two example standards (solid lines) with all DEIM points (vertical dashed lines). (b) For visualization purposes, a selected subregion about the Fe absorption edges for the set of Fe spectra and the DEIM points within this region and (c) the corresponding PCA components, along with the (d) explained variance ratio of these PCA components and the order of DEIM point selection.

To demonstrate how the DEIM points and approximation of spectra work in practice, an example study involving bulk Fe XANES measurements will be used. The description of the system used, in this case, is based on a database of Fe XANES measured from a range of 12 known standards. An additional flat spectrum, set to 10^–8^ to represent a near-zero background, is also added to the set to provide a total of 13 spectra. These standards were measured as part of an effort to build a database of standards on the core EXAFS beamline B18 at Diamond.^15,16^ As a demonstration of the workings of the method for these ideal spectra, Figure 2 shows the spectra, the extracted PCA components from the spectra, and the 13 sampling points selected by the DEIM method.

The DEIM algorithm chooses points based on the magnitude of a residual, which depends on the changes from successive PCA components, so it is dependent on the spectra used and naturally weights toward feature changes, which occur particularly about the absorption edge, resulting in grouping of DEIM points around this region. The order in which the DEIM points are chosen is displayed within the plot of the explained variance for each component (Figure 2d).

To demonstrate the technique in an experimental microscopy context, Fe_2_O_3_ and Fe_3_O_4_ powders were ground and drop-cast onto a silicon nitride membrane. X-ray fluorescence (XRF) XANES mapping measurements using a 50 nm beam probe, a 50 nm scan pixel size, and a 15 ms dwell time per point were conducted on beamline I14^17^ at Diamond Light Source. A full spectro-microscopy map was collected as a ground truth measurement over a 5 × 5 μm region. The full scan used 152 energies with a 10 eV step in the pre-edge, 0.5 eV steps in a region from −20 to +30 eV about the absorption edge, and a gradually increasing energy step in the post-edge. The DEIM method was used to sample a larger region with 13 energy points selected based on the previously described dictionary of spectra. The reduced sampling of the DEIM approach allowed, in this case, for a wider field of view of 20 × 20 μm to be measured in similar time scales to the full measurement. The full measurement consists of 10 000 individual pixels with a spectrum per pixel. Manual interrogation of data sets of this scale is challenging, so cluster analysis is typically used to differentiate and group similar spectra within spectro-microscopy data sets.^18,19^ Cluster analysis also allows for statistical averaging, improving the signal-to-noise ratio, as the extracted spectra are from the average of all spectra in a cluster. The optimal number of clusters is determined manually or through the use of the gap statistic^20^ or silhouette analysis.^21^ In this case, three clusters were determined to be sufficient.

**Figure 3:**
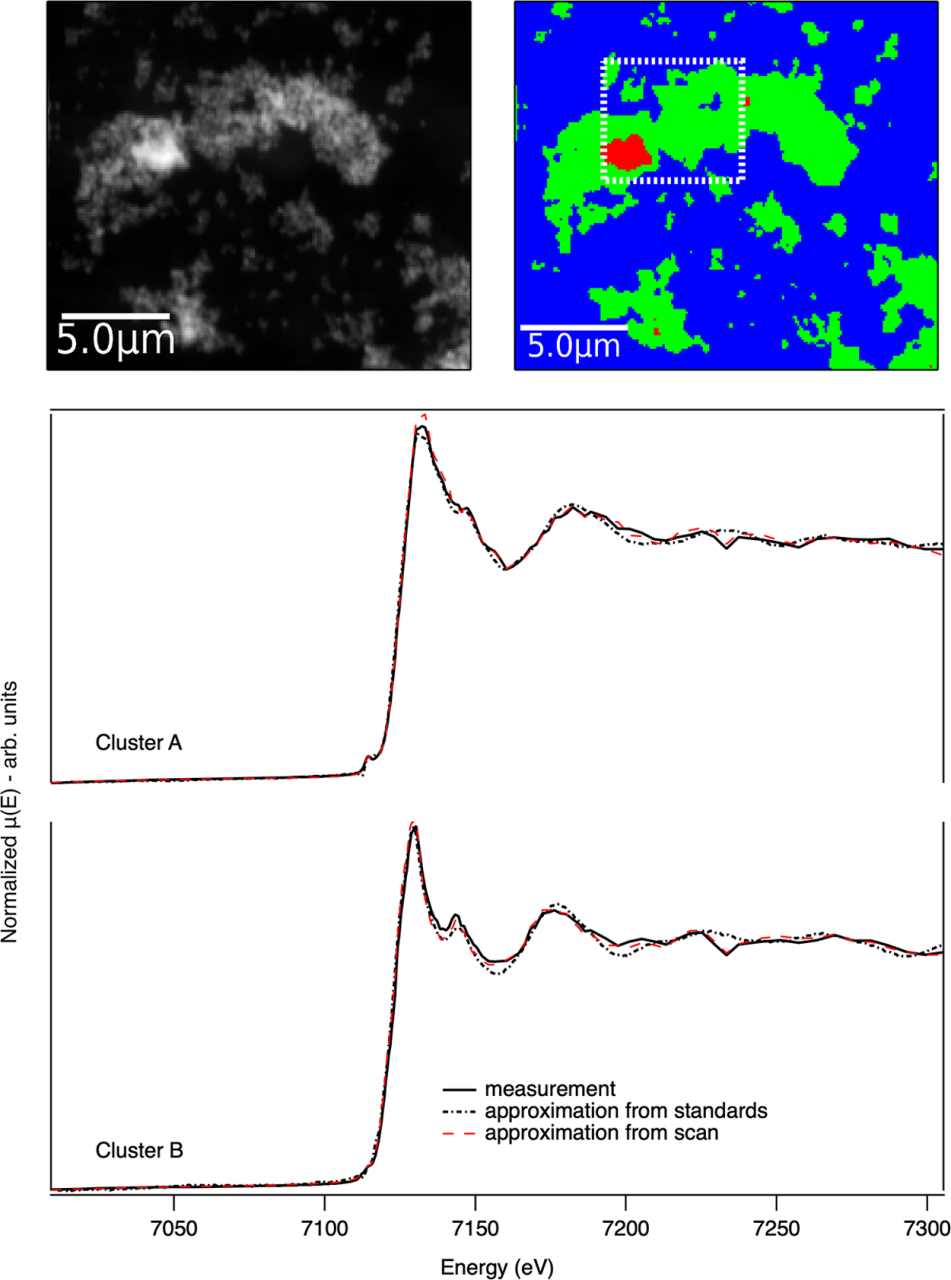
X-ray spectro-microscopy results from the Fe K-edge of a Fe_2_O_3_ and Fe_3_O_4_ test sample. (Top left) XRF map of the Fe distribution in the sample and (top right) corresponding cluster analysis map showing regions with similar spectral (XANES) measurements. The dashed-square in the image indicates the region measured by using a full XANES measurement. A wider scan area was measured using DEIM sampling. (Bottom) Comparison of the Fe XANES spectra corresponding to the green and red cluster regions, respectively, from the full measurement and the DEIM approximations generated using standards or the previous full scan data.

The methodology of extracting the approximated spectra is like that of the full scan case. The DEIM approximation is not applied pixel by pixel; rather, the DEIM energy points are grouped using cluster analysis to benefit from a better signal-to-noise ratio by statistical averaging. The resulting cluster centers are used to solve for *C* as per eq 2. Two variants of the approximation using DEIM were examined. The first used the PCA components of the Fe reference spectra for the approximation, and the second used the PCA components from the full spectro-microscopy scan. A representative XRF map from the DEIM scans, the resulting cluster map, and the extracted XANES spectra are shown in Figure 3.

Both of the approximations using the information from the standards and the full scan clearly capture the chemical states present both in the structure and edge position. The approximation based on information from the full scan results in better matching to features, even replicating an intensity drop at 7230 eV. It should be noted, however, that the comparison and how the approximation is obtained are not identical. The full scan only has a small number of chemical states, so the number of PCA components needed is lower than in the case of the 13 reference spectra. The strategy of using sampling points based on the richer set of reference spectra provides the flexibility to move between both reference standards and data or to combine both as an experiment progresses.

The previous examples, while demonstrating the method, did not explicitly treat or include some of the experimental variances that might occur, such as background variations and the potential impact of noise on the measurements and the resulting DEIM approximation.

### Background Variations

To improve the accuracy of the approximation further, a reduced description and sampling can be developed, which describes both the spectra of interest and any background variations. X-ray absorption data reduction typically requires a first- or second-order polynomial to be fitted to the pre-edge region. In the post-edge region for EXAFS, the background variation is approximated with a piecewise polynomial or spline but with the objective to extract the EXAFS signal and minimize low-frequency components.^22^ For XANES, functions incorporating second- or third-order Legendre polynomials have been shown to be sufficient in fitting or describing post-edge background variations.^23^

To incorporate these polynomial variations, a diverse set of spectra with a range of background variations was created. PCA was then applied to obtain a set of orthogonal components that provides a reduced description of the set of standards and background variations. Specifically, for each XAS standard, 500 random variations were created, resulting in a total of 6500 spectra. Each XAS standard was augmented with Bézier curves to produce the background variants. A fixed control point is used before the edge for the pre-edge background and just after the edge for the post-edge background. To create each background variant, control points are randomly chosen in energy within the pre- and post-edge region, respectively, and randomly offset, within some range, about the value at that energy point. The pre-edge background was extended and applied over the full spectrum, while the post-edge background was only applied after the absorption edge. The DEIM approach reduces this set of spectra to *N* + 2 components and sampling points in the case of a quadratic background and *N* + 5 for the most general case of a quadratic background and a cubic post-edge.

This approach of generating a set of background-adjusted spectra allows for constraints to be applied to the range of background variations in the pre- and post-edge regions and the flexible incorporation of backgrounds within specific ranges of the spectra.

**Figure 4:**
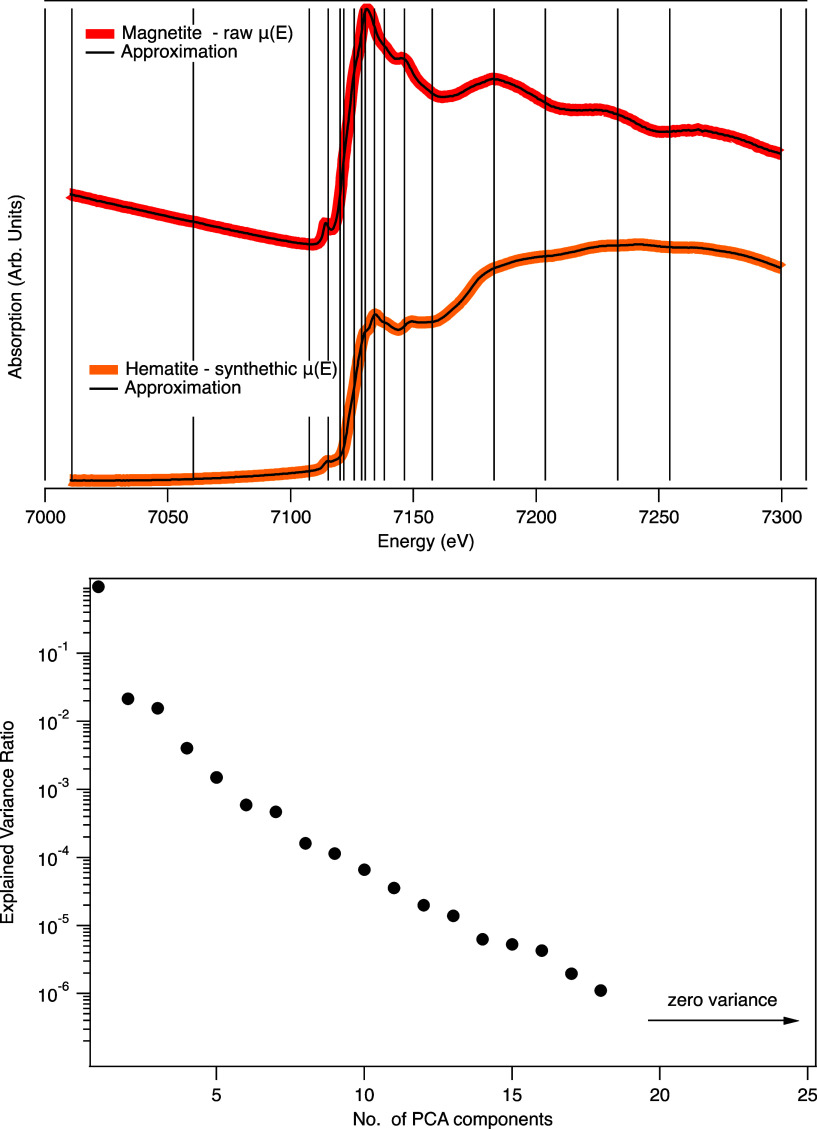
Example of using the DEIM approximation with background variations. Top: DEIM approximations to a raw magnetite Fe XAS spectrum and to a synthetically modified Hematite spectrum. Bottom: the explained variance of the PCA components created from the set of Fe spectra with randomly generated background polynomials, showing the quadratic pre-edge and cubic post-edge background variations are reduced to five additional components.

The incorporated background approach was tested against real and synthetically generated spectra. Examples of the approximations of a raw, unprocessed Fe magnetite XAS spectrum and a synthetically modified hematite spectrum are shown in Figure 4.

The approximations required 18 sampling points to accurately recover the original spectra. The explained variance from the PCA of the background variation data set (Figure 4) demonstrates that an orthogonal description of the background polynomials is retrieved via PCA and that the quadratic pre-edge and cubic post-edge variations within the full set of 6500 spectra (and more generally) can be explained with five additional components.

Note that the background components are not separable from the spectral components within the approximation, and conventional XAS processing would still need to be applied to the resulting approximation to produce a normalized result.

### Noise and Oversampling

The approximation accuracy can be reduced due to the limited number of sampling points affecting the estimate of *C* (eq 2) when data is noisy. The sampling points, as shown in Figure 1, also do not sample the pre- and post-edge regions extensively, so the match between the data and approximation can also be weaker at these points as a result. To further improve robustness and accuracy, greedy approaches can be used, in which additional oversampling can improve stability and accuracy.^24^ These greedy approaches have taken the form of additional random samples or structured sampling weighting lower-weighted components. The distinct features and regions of the XAS spectra allow for the DEIM method to be applied to specific regions or intervals within the spectra to provide sampling points that emphasize features in each region, and the resulting sampling points from each region are then aggregated. This was preferred, as it was more intuitive with clearer scaling properties compared to random sampling. The effect on accuracy and the degree to which any oversampling or subdivision is needed will depend on the level of noise, the number of chemical states, and how distinct they are. This can be effectively simulated prior to an experiment, but in practical tests, the DEIM method was sufficient to a few percent noise with additional oversampling required above ±1–2% noise. The choice of whether to oversample or modify the DEIM measurements to improve the signal-to-noise ratio can also be considered.

**Figure 5:**
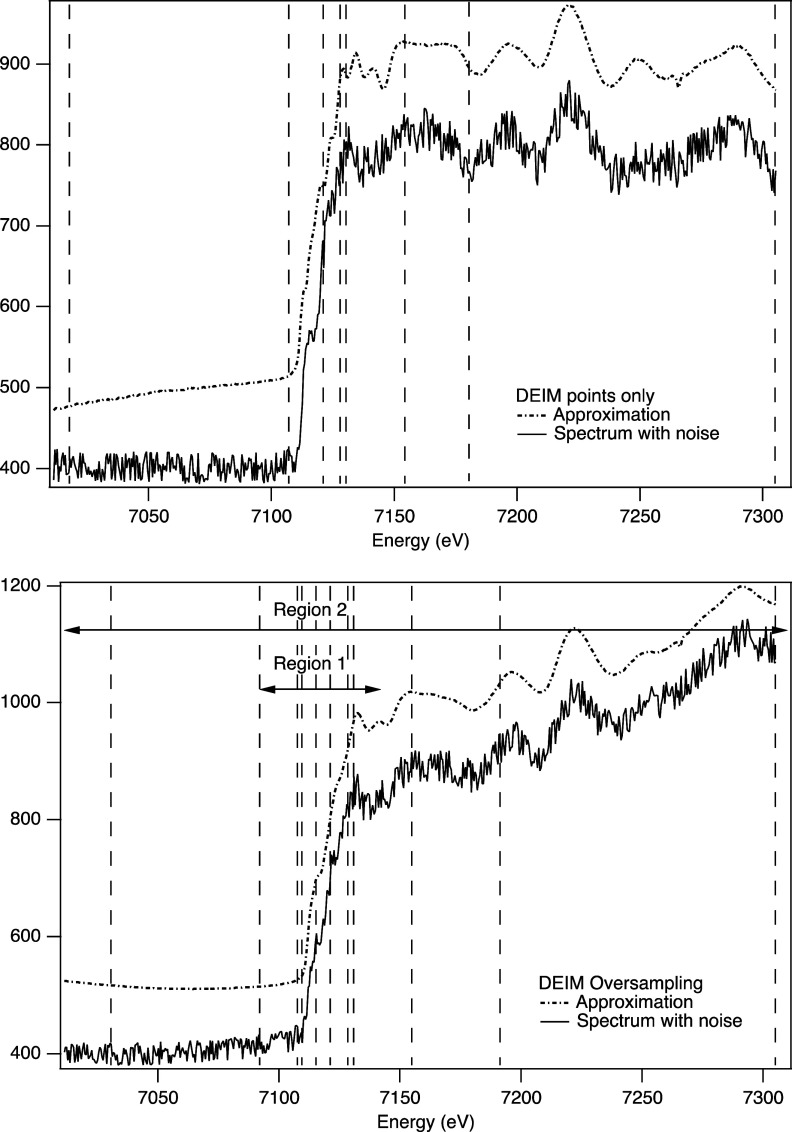
Example of using the DEIM approximation with noisy data. The approximation is offset vertically to aid in comparison. Top: a selected example of a poor approximation using standard DEIM sampling based on four XAS spectral standards with background variations, resulting in seven sampling points. Bottom: a selected example using oversampling, achieved by applying DEIM to the edge region in isolation and combining with the standard DEIM estimation across the full range. This resulted in 11 unique points rather than 14 (two regions with seven DEIM points per region) due to some duplication, which is to be expected.

The performance of DEIM for a Fe metal XANES spectrum with ±4% noise for this four-spectra case and the use of oversampling are shown in Figure 5.

In this case, the approximation from the standard DEIM approach can sometimes result in inaccurate approximations. The example shown is of a worst-case approximation, selected from a series of tests, which has some sharp steps and features about the edge step that are not present in a standard Fe metal XAS spectrum. To improve accuracy, the DEIM points were oversampled by combining the DEIM points estimated from the total spectra and those from applying DEIM to a small region at the edge. This resulted in some duplication of sampling points, resulting in 11 unique sampling points. Across a series of tests, this additional sampling improved the approximation and accurately replicated the underlying spectrum of the measurement while also approximating the background variation.

## Discussion

The application of the DEIM approach inherently relies on a good energy calibration between the active measurement and the reference spectra and good reproducibility in the energy position during the XAS measurement. This requirement for reproducibility is also true for the LC method and is generally a requirement of facilities performing XAS measurements, which is considered in the design. The I18 microfocus beamline at Diamond, for example, reported a maximum shift of 0.03 eV during repeated scanning over a 36 h period.^25^ This reproducibility is much smaller than the width of the XANES features that will be determined by the resolution of the beamline (typically 10^–4^ for a Si(111) mono, or 1 eV at 10 keV) and the core hole lifetime (∼1.5 eV for a K-edge of a transition metal).^26^ In practice, if we consider measuring the intensity along a Gaussian-like feature, then an error in the sampling position of 5% of the variance would have very little effect around the peak but would result in up to a 3% intensity change at the inflection point. A coarse estimate of the reproducibility requirement for energy sampling methods would, therefore, be 5–10% of the feature width. In situations where drift is a concern, the simultaneous measurement of a reference standard can sometimes be incorporated to provide an internal calibration for all measurements.^27^

How measurement errors affect the approximation from DEIM will depend on the system, the number of reference spectra or PCA components, and the feature changes at sampling points. If the PCA components in the pre-edge region, for example, are all flat, then the approximation will be flat in this region regardless of noise or error in position. As with the noise example, these tolerances can all be readily tested and simulated when designing an experiment.

The DEIM method described here has recently been used in the study of Pt complexes for use in photoactivated chemotherapy.^28^ These complexes are inert in the dark but release Pt(II) species and radicals upon visible light irradiation, resulting in photocytotoxicity toward cancer cells. The concentrations present in the cells would result in very long acquisition times, making conventional spectro-microscopy very challenging due to the accumulated damage and dose in the surrounding cell. XANES measurements using the DEIM approach were used to image Pt^4+^ and Pt^2+^ and showed that cells treated with Pt^2+^ only partially reduce upon irradiation, showing both the value of this approach and that X-ray induced photoactivated redox could be avoided.

**Figure 6:**
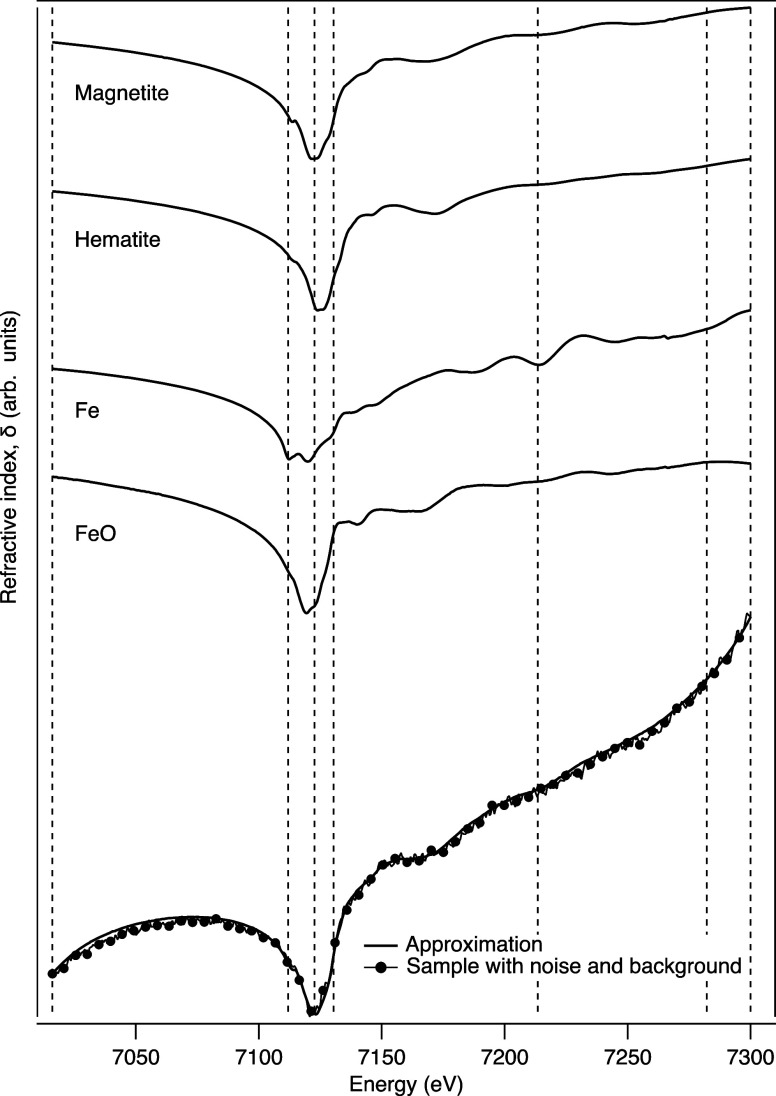
Example of the application of the DEIM approach to refractive index measurements over an absorption edge. The figure shows the Kramers–Kronig transform of four absorption spectra and an example of the DEIM sampling points. Background variations and noise were also included, and a comparison of a noisy, varying spectrum with an approximation determined from seven DEIM points samples shows very good agreement.

As the DEIM approach is based on building a low-rank description of a system, it can be applied to any experimental spectral or spectro-microscopy measurement where PCA or a similar approach can be applied to the previous measurements or standard measurement data. To show a more general application of the approach, phase spectra were constructed from the Kramers–Kronig transform of a set of four Fe XAS absorption spectra. The DEIM approach was applied to phase spectra with a quadratic background variation only applied to the XAS, as per the previous descriptions, to extract the DEIM points and components. A randomly selected phase spectrum with a background variation and noise (<1%) applied was then sampled at the DEIM points and approximated for comparison (Figure 6).

As the DEIM algorithm chooses points based on the magnitude of a residual, which depends on the changes between successive PCA components, the sampling points group around large changes, which typically occur near the absorption edge. This results in a limited sampling of weaker pre-edge and post-edge (Figure 3). If the pre-edge is a critical feature to approximate accurately, the approach used for the noisy data example of applying DEIM to sections of interest and aggregating DEIM points to increase sampling in those areas could be used, although this will increase the overall number of sampling points. A possible alternative to improve the sampling of weaker features is to change the DEIM approach from using the maximum of the residual to decide on the sampling points to another metric, such as the maximum significant discrepancy. This may help improve the sampling and the resulting approximation in the pre-edge and post-edge regions, but this has not yet been evaluated and will be the subject of future work.

## Conclusions

We have presented a method to approximate XAS spectra using a greatly reduced number of energy points based on a low-rank description formed from a set of conveniently measured XAS spectra or previous spectro-microscopy scans. This approach allows for the design of optimized imaging experiments that reduce experiment time and dose and that can incorporate noise and background effects, treatments for which have previously limited the accuracy and application of reduced sampling approaches. This method has applications in spectro-microscopy and flux-hungry XAS experiments, where knowledge of the possible chemical states of the system is available and a faster or lower-dose measurement is required. The technique can be used to rapidly screen samples for chemical state variations when spatial mapping or improved temporal capabilities for *in situ* measurements is needed. The method can also be used to address challenging samples where measurements have, to date, been limited due to dose sensitivity of the sample or longer collection times resulting from low concentrations within the sample and provides a solid mathematical basis for selecting sampling points and reducing the number of measurement points. The ability to develop and adapt the low-rank description of the system will improve as XAS databases become more readily available. Improvements to the approximation should be possible by the inclusion of more advanced noise and likelihood-based modeling. The approach potentially lends itself to adaptive approaches either to measure a mixture of sparse and full data sets to improve approximation or to hybrid machine learning approaches to adaptively adjust sampling during an experiment.

## References

[ref1] Yu Y.-S.; Farmand M.; Kim C.; Liu Y.; Grey C. P.; Strobridge F. C.; Tyliszczak T.; Celestre R.; Denes P.; Joseph J.; Krishnan H.; Maia F. R. N. C.; Kilcoyne A. L. D.; Marchesini S.; Leite T. P. C.; Warwick T.; Padmore H.; Cabana J.; Shapiro D. A. Three-Dimensional Localization of Nanoscale Battery Reactions Using Soft X-Ray Tomography. Nat. Commun. 2018, 9 (1), 921 10.1038/s41467-018-03401-x. 29500344 PMC5834601

[ref2] Wise A. M.; Weker J. N.; Kalirai S.; Farmand M.; Shapiro D. A.; Meirer F.; Weckhuysen B. M. Nanoscale Chemical Imaging of an Individual Catalyst Particle with Soft X-Ray Ptychography. ACS Catal. 2016, 6 (4), 2178–2181. 10.1021/acscatal.6b00221. 27076990 PMC4822187

[ref3] Baier S.; Damsgaard C. D.; Scholz M.; Benzi F.; Rochet A.; Hoppe R.; Scherer T.; Shi J.; Wittstock A.; Weinhausen B.; Wagner J. B.; Schroer C. G.; Grunwaldt J.-D. In Situ Ptychography of Heterogeneous Catalysts Using Hard X-Rays: High Resolution Imaging at Ambient Pressure and Elevated Temperature. Microscopy and Microanalysis 2016, 22 (1), 178–188. 10.1017/S1431927615015573. 26914998

[ref4] Jacobsen C. J.; Wang S. Y.; Yun W.; Frigo S. Calculation of X-Ray Refraction from near-Edge Absorption Data Only. In Optical Constants of Materials for UV to X-Ray Wavelengths; Soufli R., Seely J. F., Eds.; SPIE, Denver, Colorado, U.S., 2004; Vol. 5538, p 23–30. 10.1117/12.560160.

[ref5] Boesenberg U.; Ryan C. G.; Kirkham R.; Jahn A.; Madsen A.; Moorhead G.; Falkenberg G.; Garrevoet J. Fast XANES Fluorescence Imaging Using a Maia Detector. J. Synchrotron Rad 2018, 25 (3), 892–898. 10.1107/S1600577518004940. 29714202

[ref6] Kandel S.; Zhou T.; Babu A. V.; Di Z.; Li X.; Ma X.; Holt M.; Miceli A.; Phatak C.; Cherukara M. J. Demonstration of an AI-Driven Workflow for Autonomous High-Resolution Scanning Microscopy. Nat. Commun. 2023, 14 (1), 5501 10.1038/s41467-023-40339-1. 37679317 PMC10485018

[ref7] Townsend O.; Gazzola S.; Dolgov S.; Quinn P. Undersampling Raster Scans in Spectromicroscopy for a Reduced Dose and Faster Measurements. Opt. Express 2022, 30 (24), 43237 10.1364/OE.471663. 36523026

[ref8] Cohen S. X.; Webb S. M.; Gueriau P.; Curis E.; Bertrand L. Robust Framework and Software Implementation for Fast Speciation Mapping. J. Synchrotron Rad 2020, 27 (4), 1049–1058. 10.1107/S1600577520005822. 33566015

[ref9] Gomez-Gonzalez M. A.; Koronfel M. A.; Goode A. E.; Al-Ejji M.; Voulvoulis N.; Parker J. E.; Quinn P. D.; Scott T. B.; Xie F.; Yallop M. L.; Porter A. E.; Ryan M. P. Spatially Resolved Dissolution and Speciation Changes of ZnO Nanorods during Short-Term in Situ Incubation in a Simulated Wastewater Environment. ACS Nano 2019, 13 (10), 11049–11061. 10.1021/acsnano.9b02866. 31525960

[ref10] Nowack L.; Grolimund D.; Samson V.; Marone F.; Wood V. Rapid Mapping of Lithiation Dynamics in Transition Metal Oxide Particles with Operando X-Ray Absorption Spectroscopy. Sci. Rep. 2016, 6 (1), 21479 10.1038/srep21479. 26908198 PMC4764840

[ref11] Wang J.; Chen-Wiegart Y.-c. K.; Wang J. In Operando Tracking Phase Transformation Evolution of Lithium Iron Phosphate with Hard X-Ray Microscopy. Nat. Commun. 2014, 5 (1), 4570 10.1038/ncomms5570. 25087693

[ref12] Chaturantabut S.; Sorensen D. C. Nonlinear Model Reduction via Discrete Empirical Interpolation. SIAM Journal on Scientific Computing 2010, 32 (5), 2737–2764. 10.1137/090766498.

[ref13] Antoulas A. C.; Sorensen D. C. Approximation of Large-Scale Dynamical Systems: An Overview. Int. J. Appl. Math. Comput. Sci. 2001, 11 (5), 1093–1121.

[ref14] Satopaa V.; Albrecht J.; Irwin D.; Raghavan B. Finding a “Kneedle” in a Haystack: Detecting Knee Points in System Behavior. In 2011 31st International Conference on Distributed Computing Systems Workshops; IEEE, Minneapolis, Minnesota, U.S., 2011; p 166–171. 10.1109/ICDCSW.2011.20.

[ref15] Dent A. J.; Cibin G.; Ramos S.; Parry S. A.; Gianolio D.; Smith A. D.; Scott S. M.; Varandas L.; Patel S.; Pearson M. R.; Hudson L.; Krumpa N. A.; Marsch A. S.; Robbins P. E. Performance of B18, the Core EXAFS Bending Magnet Beamline at Diamond. Journal of Physics: Conference Series 2013, 430 (1), 012023 10.1088/1742-6596/430/1/012023.

[ref16] Cibin G.; Gianolio D.; Parry S. A.; Schoonjans T.; Moore O.; Draper R.; Miller L. A.; Thoma A.; Doswell C. L.; Graham A. An Open Access, Integrated XAS Data Repository at Diamond Light Source. Radiat. Phys. Chem. 2020, 175, 108479 10.1016/j.radphyschem.2019.108479.

[ref17] Quinn P. D.; Alianelli L.; Gomez-Gonzalez M.; Mahoney D.; Cacho-Nerin F.; Peach A.; Parker J. E. The Hard X-Ray Nanoprobe Beamline at Diamond Light Source. Journal of Synchrotron Radiation 2021, 28 (3), 1006–1013. 10.1107/S1600577521002502. 33950009 PMC8127369

[ref18] Lerotic M.; Jacobsen C.; Schäfer T.; Vogt S. Cluster Analysis of Soft X-Ray Spectromicroscopy Data. Ultramicroscopy 2004, 100 (1–2), 35–57. 10.1016/j.ultramic.2004.01.008. 15219691

[ref19] Lerotic M.; Mak R.; Wirick S.; Meirer F.; Jacobsen C. Mantis: A Program for the Analysis of x-Ray Spectromicroscopy Data. Journal of Synchrotron Radiation 2014, 21 (5), 1206–1212. 10.1107/S1600577514013964. 25178014

[ref20] Tibshirani R.; Walther G.; Hastie T. Estimating the Number of Clusters in a Data Set Via the Gap Statistic. Journal of the Royal Statistical Society Series B: Statistical Methodology 2001, 63 (2), 411–423. 10.1111/1467-9868.00293.

[ref21] Rousseeuw P. J. Silhouettes: A Graphical Aid to the Interpretation and Validation of Cluster Analysis. Journal of Computational and Applied Mathematics 1987, 20, 53–65. 10.1016/0377-0427(87)90125-7.

[ref22] Newville M.; Li̅viņš P.; Yacoby Y.; Rehr J. J.; Stern E. A. Near-Edge x-Ray-Absorption Fine Structure of Pb: A Comparison of Theory and Experiment. Phys. Rev. B 1993, 47 (21), 14126–14131. 10.1103/PhysRevB.47.14126. 10005753

[ref23] Weng T.-C.; Waldo G. S.; Penner-Hahn J. E. A Method for Normalization of X-Ray Absorption Spectra. J. Synchrotron Rad 2005, 12 (4), 506–510. 10.1107/S0909049504034193. 15968130

[ref24] Peherstorfer B.; Drmač Z.; Gugercin S. Stability of Discrete Emprical Interpolation and Gappy Proper Orthogonal Decomposition with Randomized and Deterministic Sampling Points. SIAM Journal on Scientific Computing 2020, 42 (5), A2837–A2864. 10.1137/19M1307391.

[ref25] Mosselmans J. F. W.; Quinn P. D.; Dent A. J.; Cavill S. A.; Moreno S. D.; Peach A.; Leicester P. J.; Keylock S. J.; Gregory S. R.; Atkinson K. D.; Rosell J. R. I18 – the Microfocus Spectroscopy Beamline at the Diamond Light Source. J. Synchrotron Rad 2009, 16 (6), 818–824. 10.1107/S0909049509032282. 19844019

[ref26] Krause M. O.; Oliver J. H. Natural Widths of Atomic K and L Levels, Kα X-ray Lines and Several KLL Auger Lines. J. Phys. Chem. Ref. Data 1979, 8 (2), 329–338. 10.1063/1.555595.

[ref27] Cross J. O.; Frenkel A. I. Use of Scattered Radiation for Absolute X-Ray Energy Calibration. Rev. Sci. Instrum. 1999, 70 (1), 38–40. 10.1063/1.1149539.

[ref28] Bolitho E. M.; Sanchez-Cano C.; Shi H.; Quinn P. D.; Harkiolaki M.; Imberti C.; Sadler P. J. Single-Cell Chemistry of Photoactivatable Platinum Anticancer Complexes. J. Am. Chem. Soc. 2021, 143 (48), 20224–20240. 10.1021/jacs.1c08630. 34808054 PMC8662725

